# Isolation of a *Tissierellaceae* Bacterium Exhibiting a High Reduction Potential for Insoluble Indigo Dyes

**DOI:** 10.1264/jsme2.ME24104

**Published:** 2025-07-12

**Authors:** Zhihao Tu, Isao Yumoto

**Affiliations:** 1 MOE Key Laboratory of Groundwater Circulation and Evolution & School of Water Resources and Environment, China University of Geosciences (Beijing), No. 29, Xueyuan Road, Haidian District, Beijing 100083, PR China; 2 Institute for Open and Transdisciplinary Research Initiatives, Osaka University, 2–1 Yamada-oka, Osaka, 565–0871, Japan

**Keywords:** traditional indigo dyeing, indigo reduction, isolation, *Tissierellaceae*, electron mediators

## Abstract

In traditional indigo dyeing, water-insoluble indigo is anaerobically converted into soluble leuco-indigo via microbial reduction in alkaline dye suspensions, allowing its use as a fabric dye. Although various indigo-reducing bacteria have been isolated to date, culture-independent microbial community ana­lyses have suggested that bacteria belonging to uncultured clades also contribute to indigo reduction. Therefore, we aimed to isolate previously overlooked indigo-reducing bacteria using an unconventional culture method. We conducted enrichment cultures and single-colony isolation using a medium supplemented with *sukumo*, an indigo dye source derived from the composted leaves of indigo-containing plants, as the sole energy, carbon, and nitrogen sources. We isolated a previously uncultured bacterium belonging to the family *Tissierellaceae*, which had been predicted as a major indigo reducer in various indigo dyeing processes solely based on microbial community ana­lyses. The insoluble indigo-reducing activity of the *Tissierellaceae* isolate, strain TU-1 was significantly higher than that of known indigo-reducing bacteria. The addition of the culture supernatant of strain TU-1 enhanced the reduction of indigo powder by other indigo-reducing bacteria, with similar stimulatory effects to those of the insoluble electron mediator, anthraquinone. These results indicate that strain TU-1 possesses a high capacity for secreting electron mediators, conferring a significant reduction capacity for insoluble indigo. Further investigations, including the discovery of additional unknown indigo-reducing bacteria and the identification of the mediators they produce, will provide a more detailed understanding of the mechanisms underlying indigo reduction in practical dyeing processes.

*Aizome*, the traditional method for indigo dyeing in Japan, has been deeply integrated into Japanese culture, as evidenced by the term “Japan blue” (Ueno, 2023). As observed in other bioprocesses, such as fermented food production, the functions of microorganisms within their characteristic communities are effectively harnessed in traditional indigo dyeing (Aino *et al.*, 2010). In traditional indigo dyeing, leaves from plants, such as *Polygonum tinctorium*, which contain indican (indoxyl-β-D-glucoside: a precursor of indigo), undergo microbial fermentation for 3–4 months to produce a dye material known as *sukumo*. *Sukumo* is suspended in water along with wood ash, slaked lime, and microbial nutrients, such as wheat bran, which undergoes microbial fermentation for 1–2‍ ‍weeks to produce dye suspensions. Since *sukumo* and active dye suspensions are highly alkaline (pH>10), the indigo dyeing processes are primarily facilitated by alkaliphilic bacteria. Indigo is water-insoluble in dye suspensions; therefore, it needs to be reduced under anaerobic conditions to its soluble form, leuco-indigo, in order to function as a fabric dye. Indigo reduction in dye suspensions is facilitated by certain alkaliphilic bacteria, specifically known as indigo-reducing bacteria. Indigo fermentation fluid holds the indigo reduction state for more than 6 months. High pH and anaerobic conditions prevent external contamination. It also requires the high robustness of a nutrient cycle that sustains the metabolism of microorganisms, including extracellular electron transfer involving bacteria.

A diverse array of microorganisms are present in indigo dye suspensions (Aino *et al.*, 2010, 2018; Milanović *et al.*, 2017), among which indigo-reducing bacteria are the most functionally important members. Several research groups, including ours, have isolated various indigo-reducing bacteria, most of which are facultatively anaerobic alkaliphiles belonging to the class *Bacilli* and include the genera *Alkalibacterium* (Yumoto *et al.*, 2004, 2008; Nakajima *et al.*, 2005), *Amphibacillus* (Hirota *et al.*, 2013a, 2013c), *Bacillus* (Aino *et al.*, 2008; Hirota *et al.*, 2018), *Fermentibacillus* (Hirota *et al.*, 2016a), *Fundicoccus* (Tu *et al.*, 2022), *Oceanobacillus* (Hirota *et al.*, 2013b, 2013d), *Paralkalibacillus* (Hirota *et al.*, 2017), and *Polygonibacillus* (Hirota *et al.*, 2016b). These indigo-reducing bacteria have been isolated using alkaline nutritionally rich media containing sugars and peptides as the substrates, with the reduction of indigo carmine (indigo-5,5ʹ-disulfonic acid: a soluble derivative of indigo) serving as the reduction indicator. However, microbial community ana­lyses of indigo dye suspensions have suggested that more phylogenetically diverse bacteria, which remain uncultured, may contribute to indigo reduction (Aino *et al.*, 2018; Tu *et al.*, 2019, 2021; Lopes *et al.*, 2021; Farjana *et al.*, 2023). These findings suggest that advances in isolation methods, such as the medium modifications and the implementation of enrichment cultures, facilitate the isolation of previously uncultured indigo-reducing bacteria. For example, a novel indigo-reducing *Pseudomonas* species, classified within *γ-proteobacteria*, was isolated through long-term enrichment cultures in an inorganic medium supplemented with glucose and indigo powder (Park *et al.*, 2012).

The present study targeted previously overlooked indigo-reducing bacteria, with a specific focus on uncultured bacteria belonging to the family *Tissierellaceae*. Microbial community ana­lyses based on 16S rRNA gene sequences revealed that the predominant sequences detected in all active indigo dye suspensions were exclusively derived from uncultured *Tissierellaceae* bacteria with sequence identities of 94–95% to *Tissierella creatinini* (Aino *et al.*, 2018; Tu *et al.*, 2019, 2021; Lopes *et al.*, 2021; Farjana *et al.*, 2023). In addition, sequences with high identities to uncultured *Tissierellaceae* bacteria were detected as predominant bacteria in alkaline iron oxide-reducing enrichment cultures (Fuller *et al.*, 2014) and food waste treatment processes where the reduction of selenate occurs (Logan *et al.*, 2023). Therefore, uncultured *Tissierellaceae* bacteria may possess the capability to reduce insoluble substances and, thus, contribute to the reduction of insoluble indigo in dye suspensions. However, *Tissierellaceae* bacteria isolated to date have been obligatory anaerobic, fermentative bacteria derived from sewage sludge, oil fields, or human and animal intestines and/or feces; there is no evidence to show that *Tissierellaceae* isolates are capable of growing in alkaline environments or of reducing indigo (Farrow *et al.*, 1995; Harms *et al.*, 1998; Alauzet *et al.*, 2014; Nazina *et al.*, 2020; Wu *et al.*, 2020; Wylensek *et al.*, 2020; Bellali *et al.*, 2022; Li *et al.*, 2022).

In the present study, we aimed to isolate *Tissierellaceae* bacteria from indigo dye suspensions by a novel culture method using *sukumo* as the sole energy, carbon, and nitrogen sources. The isolate obtained, along with known indigo-reducing bacteria, were assessed for their capacity to reduce insoluble indigo. The effects of electron mediator compounds and the culture supernatant of the *Tissierellaceae* isolate on the insoluble indigo reduction activity of the isolate and known indigo reducers were investigated, and the results obtained will help elucidate the mechanisms by which insoluble indigo is reduced.

## Materials and Methods

### Isolation and culture of indigo-reducing bacteria

*Sukumo*-based fermentation fluid (Batch 1, Tu *et al.*, 2021) was prepared by our group and used as the isolation source. Glycerol was added to the fermentation fluid immediately after sampling to a final concentration of 25% (v/v) and stored at –80°C until used. The alkaline *sukumo* medium was prepared as follows: vials (capacity of 68‍ ‍mL) were filled with 18‍ ‍mL of a *sukumo* suspension (76‍ ‍g‍ ‍L^–1^), flushed with N_2_ gas, and sealed with butyl rubber stoppers and aluminum caps before autoclaving. After autoclaving (121°C, 20‍ ‍min), 2‍ ‍mL of 1 M Na_2_CO_3_/NaHCO_3_ buffer (pH 10) was injected into the vials in an anaerobic chamber (Vinyl Anaerobic Chambers Type C; Coy Laboratory Products). One milliliter of the fermentation fluid was inoculated into the alkaline *sukumo* medium following an incubation at 27°C without shaking for 4 days. After four repetitions of the enrichment cultures, serial dilutions of the culture solution were spread onto the supernatant of alkaline *sukumo* medium supplemented with 2‍ ‍g‍ ‍L^–1^ indigo carmine (Fujifilm Wako Pure Chemical), and solidified with 10‍ ‍g‍ ‍L^–1^ gellan gum. The inoculated plates were incubated under anaerobic conditions using an AnaeroPack pouch bag with an AnaeroPack oxygen absorber (Mitsubishi Gas Chemical) at 27°C for 10 days. Colonies that formed clear zones due to indigo carmine reduction were picked up and purified using a peptone/yeast extract/alkaline (PYA) agar medium (Hirota *et al.*, 2013c). Following purification, the 16S rRNA gene sequences of the isolates were elucidated as described below. One of the isolates, named strain TU-1, with a sequence closely related to the target (uncultured *Tissierellaceae*) was subjected to further study. Strain TU-1 has been deposited to the Japan Collection of Microorganisms (JCM), RIKEN-BRC, under the deposition number JCM 37169.

Strain TU-1 was routinely cultured in PYA medium under anaerobic conditions at 30°C without shaking. To evaluate of its capacity for iron oxide reduction, strain TU-1 was anaerobically cultured in PYA medium supplemented with 1‍ ‍mM of poorly crystalline iron oxides. Poorly crystalline iron oxides were synthesized by adjusting a 36‍ ‍g‍ ‍L^–1^ FeCl_3_·6H_2_O solution to pH 7 using 5N NaOH, followed by washing of the resulting precipitate with distilled water (Lovley and Phillips, 1986). *Amphibacillus indicireducens* JCM 17250^T^ (Hirota *et al.*, 2013a), *Amphibacillus iburiensis* JCM 18529^T^ (Hirota *et al.*, 2013c), *Alkalibacterium iburiense* JCM 12662^T^ (Nakajima *et al.*, 2005), and *Fundicoccus fermenti* JCM 34140^T^ (Tu *et al.*, 2022) were used as model strains of indigo-reducing bacteria. These strains were routinely cultured in PYA medium under aerobic and anaerobic conditions at 30°C with (180‍ ‍rpm) and without shaking, respectively.

### Phylogenetic ana­lysis

Almost the full-length 16S rRNA gene sequence of strain TU-1 was elucidated by the direct sequencing of PCR products using the primer pair 27F/1492R as previously described (Kato *et al.*, 2004). The closest relatives of strain TU-1 were inferred using the BLAST program (Altschul *et al.*, 1997). A phylogenetic tree was constructed by the neighbor-joining method (Saitou and Nei, 1987) using the software MEGA ver. 11 (Tamura *et al.*, 2021). The bootstrap resampling method was used with 1,000 replicates to evaluate the robustness of the inferred trees (Felsenstein, 1985).

### Quantification of insoluble indigo reduction activities

In the ana­lysis of insoluble indigo reduction activities, each indigo-reducing strain was anaerobically cultured in PYA medium supplemented with 1‍ ‍g‍ ‍L^–1^ of synthetic indigo powder (Sigma-Aldrich). In the present study, insoluble indigo reduction activities were evaluated using a novel method that enables leuco-indigo concentrations to be measured in culture solutions over time. During the incubation, 500‍ ‍μL of culture fluid samples was collected using a syringe and filtered using syringe filters with a pore size of 0.45‍ ‍μm (MilliporeSigma) to remove insoluble indigo and bacterial cells. These operations were conducted under an anoxic atmosphere in an anaerobic chamber (Vinyl Anaerobic Chambers Type C) to avoid leuco-indigo oxidation. The removal of bacterial cells and indigo particles by filtration was confirmed by microscopic observations using a BX50 optical microscope (Olympus). The filtrates were vortexed under the air to oxidize leuco-indigo dissolved in the culture solution, following lyophilization to recover the oxidized product, i.e., insoluble indigo. The powder obtained was dissolved in 500‍ ‍μL of dimethyl sulfoxide (DMSO). The absorbance at 620‍ ‍nm of the DMSO solution was measured using a spectrophotometer (DeNovix DS-11; DeNovix), and indigo concentrations were measured based on a standard curve of synthetic indigo powder solution serially diluted with DMSO (Supplementary Fig. S1). The concentration of indigo in the DMSO solution corresponded to the leuco-indigo concentration in the sampled culture solution. Culture experiments were conducted in triplicate, and the Student’s *t*-test was used to assess the significance of treatment effects.

### Evaluation of effects of electron mediators

A stock solution of riboflavin (1‍ ‍g‍ ‍L^–1^) was prepared by dissolving 50‍ ‍mg of riboflavin in 50‍ ‍mL of distilled water. A stock solution of anthraquinone (0.5‍ ‍g‍ ‍L^–1^) was prepared by dissolving 20‍ ‍mg of anthraquinone in 40‍ ‍mL of DMSO. PYA media supplemented with riboflavin or anthraquinone (with final mediator concentrations of 10‍ ‍mg L^–1^) were prepared by adding 1/100 or 1/50 volumes of each stock solution, respectively, to PYA medium after autoclaving. PYA medium supplemented with DMSO (2% v/v), but not anthraquinone, was used as the control.

Culture medium supplemented with the culture supernatant of strain TU-1 (+TU-1 sup.) was prepared as follows: strain TU-1 was grown in PYA liquid medium for 3 days until reaching the early stationary phase, with a 600-nm optical density (OD_600_) of 0.08–0.10. The culture solution was centrifuged (4,500×*g*, 5‍ ‍min), and the supernatant was filtered using syringe filters (pore size, 0.22‍ ‍μm; MilliporeSigma) to obtain the spent medium. The +TU-1 sup. medium was prepared by combining 10‍ ‍mL of filtered spent medium with 10‍ ‍mL of fresh PYA medium. Indigo powder-reducing activities were measured as described above.

### Nucleotide sequence accession numbers

The nucleotide sequence data obtained from the isolates in the present study have been submitted to the DNA Data Bank of Japan (DDBJ) under the accession number LC836396.

## Results and Discussion

### Isolation of a novel indigo-reducing bacterium belonging to the family *Tissierellaceae*

Uncultured *Tissierellaceae* bacteria, which were the target of the present study, have been suggested to utilize organic compounds present in *sukumo*. This assumption is based on their notable dominance under fermentation conditions where no additional nutrients, such as wheat bran, were introduced at the onset (Tu *et al.*, 2021). In addition, sequences closely related to those of uncultured *Tissierellaceae* become dominant during the anaerobic degradation of excess sludge under alkaline conditions (pH 9–10) (Maspolim *et al.*, 2015; Huang *et al.*, 2018). These findings indicate that uncultured *Tissierellaceae* prefer to catabolize proteinaceous compounds present in *sukumo* and sludge rather than carbohydrates, which are the main component of wheat bran. Therefore, we conducted enrichment cultures in the present study using an alkaline medium supplemented with *sukumo* as the sole energy, carbon, and nitrogen sources (alkaline *sukumo* medium), followed by single colony isolation on solidified media of the same composition.

We obtained 29 isolates through culture enrichment and single colony isolation using the alkaline *sukumo* medium. We revealed through a 16S rRNA gene sequence ana­lysis that seven of these isolates shared identical sequences and may be classified within the family *Tissierellaceae*. We selected a representative strain, designated as strain TU-1, for subsequent experiments. Strain TU-1 exhibited a capacity for indigo carmine reduction under anaerobic conditions (Supplementary Fig. S2A and B). We excluded the remaining 22 isolates from further ana­lyses due to their close phylogenetic relationship with known indigo-reducing bacteria, specifically *Am. indicireducens* (Hirota *et al.*, 2013a) or *F. fermenti* (Tu *et al.*, 2022).

**Fig. 1.**
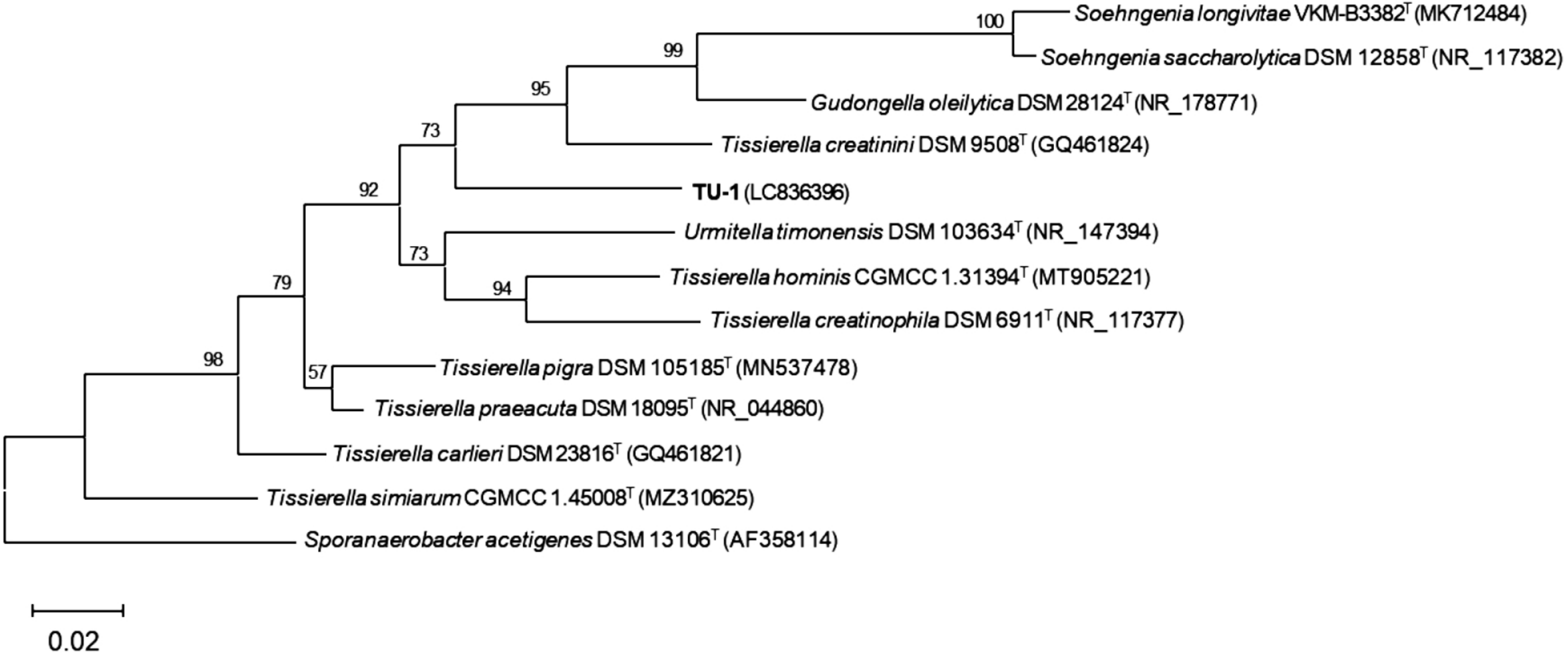
A maximum-likelihood and general time-reversible model phylogenetic tree based on 16S rRNA gene sequences of strain TU-1 and type strains in the family *Tissierellaceae*. *Sporanaerobacter acetigenes* (family *Sporanaerobacteraceae*) was used as the outgroup. Bootstrap values (1,000 trials, only >50% are shown) are indicated at branching points. Accession numbers are stated within parentheses. Bar, 0.02 substitutions per nucleotide position.

The 16S rRNA gene sequence of strain TU-1 showed a high identity to uncultured *Tissierellaceae* bacteria predominant in various indigo fermentation processes: for example, 99.3% identity to uncultured *Tissierellaceae* bacterium clone D2-9M19 (LC148765, Okamoto *et al.*, 2017). 16S rRNA gene sequence similarities with the first and second major *Tissierellaceae* sequences in Batch 1 microbiota were 99.3 and 100%, respectively (accession number: DRA011373; Tu *et al.*, 2021). The type strains closely related to strain TU-1 were *T. creatinini* DSM 9508^T^ (94.0% identity) (Farrow *et al.*, 1995) and *Tissierella pigra* DSM 105185^T^ (93.6% identity) (Wylensek *et al.*, 2020). A 16S rRNA gene-based phylogenetic tree ana­lysis suggested that strain TU-1 belonged to a cluster in the family *Tissierellaceae* (Fig. 1). These results imply that strain TU-1 represents a novel indigo-reducing bacterium within the class *Tissierellia* in the phylum *Bacillota*, while most indigo-reducing bacteria isolated to date belong to the class *Bacilli* of the phylum *Bacillota* (Yumoto *et al.*, 2004, 2008; Nakajima *et al.*, 2005; Aino *et al.*, 2008; Hirota *et al.*, 2013a, 2013b, 2013c, 2013d, 2016a, 2016b, 2017, 2018; Tu *et al.*, 2022).

### Reduction of insoluble indigo by strain TU-1

To assess the role of strain TU-1 in indigo dyeing processes, the growth properties and indigo-reducing activities of strain TU-1 and four strains known to reduce indigo were evaluated. Strain TU-1 did not grow under aerobic conditions (Supplementary Fig. S3A), suggesting that it is an obligate anaerobe, similar to other *Tissierellaceae* bacteria (Li *et al.*, 2022). Other indigo-reducing bacteria, specifically *Am. indicireducens*, *Am. iburiensis*, *Al. iburiense*, and *F. fermenti*, are reportedly facultative anaerobes (Nakajima *et al.*, 2005; Hirota *et al.*, 2013a, 2013c; Tu *et al.*, 2022), growing under both aerobic and anaerobic conditions (Supplementary Fig. S3). Under anaerobic conditions, *F. fermenti* exhibited the highest growth, reaching a maximum OD_600_ of 0.21 after 1 day of incubation. The other three known indigo-reducing bacteria reached maximum OD_600_ of 0.09–0.11 after 1–2 days of culture. Strain TU-1 required 3 days to reach the stationary phase, with a maximum OD_600_ of 0.10. Despite slight differences, anaerobic growth properties did not markedly differ between strain TU-1 and other indigo-reducing bacteria (Supplementary Fig. S3B).

**Fig. 2.**
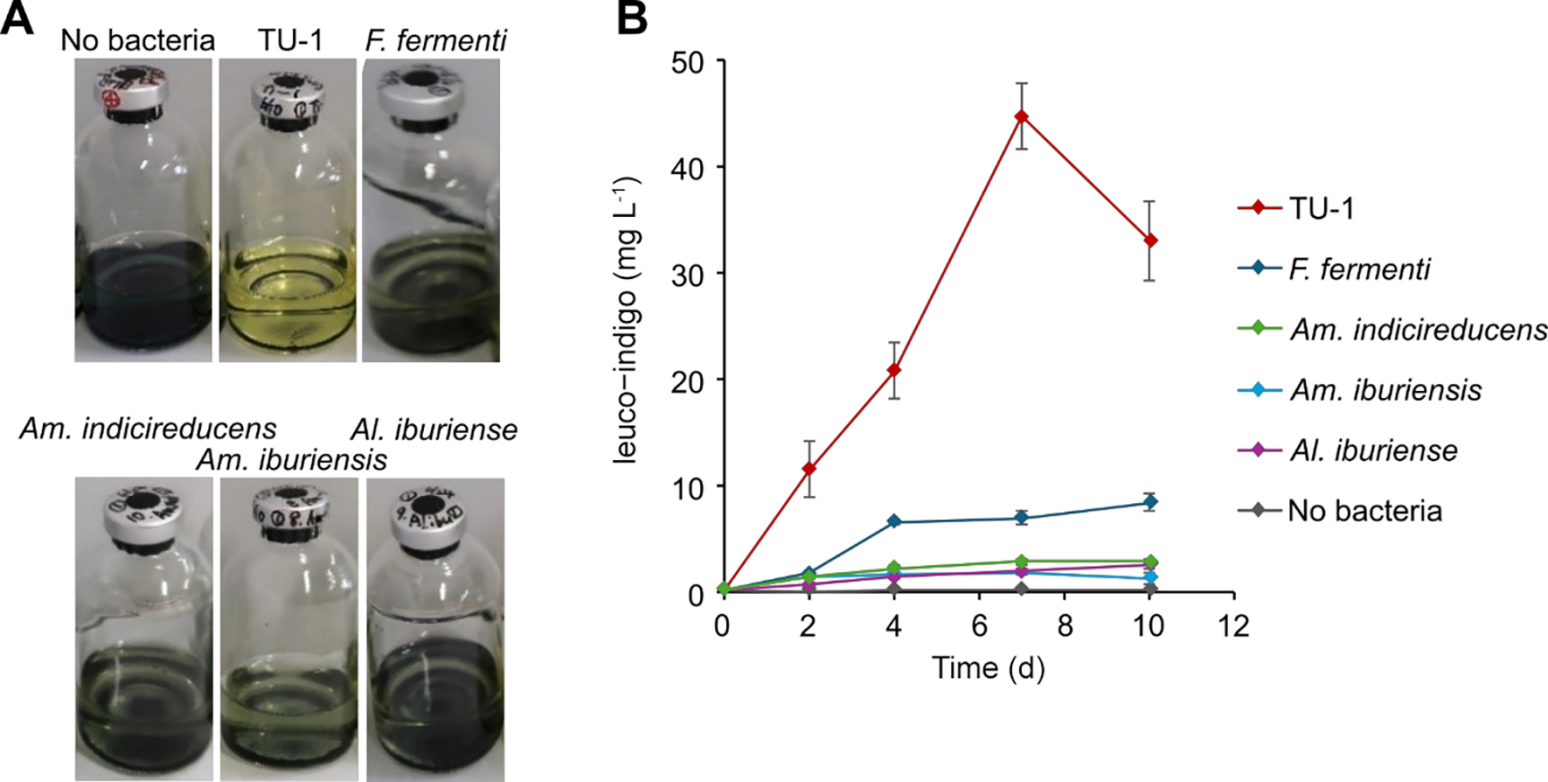
Reduction of insoluble indigo powder by strain TU-1 and known indigo-reducing bacteria. (A) Changes in color of the culture solution after 2 days of incubation. (B) The time-course of changes in the concentrations of indigo reductants (leuco-indigo) during cultures of each bacterial strain. Data are presented as the means of three independent cultures, and error bars represent standard deviations. *F. fermenti*, *Fundicoccus fermenti* JCM 34140^T^; *Am. indicireducens*, *Amphibacillus indicireducens* JCM 17250^T^; *Am. iburiensis*, *Amphibacillus iburiensis* JCM 18529^T^; *Al. iburiense*, *Alkalibacterium iburiense* JCM 12662^T^.

When strain TU-1 was cultured anaerobically in PYA medium supplemented with indigo powder, the formation of the indigo blue precipitate decreased and the culture solution turned yellowish within 2 days of incubation (Fig. 2A). This result suggests that strain TU-1 reduced powdered indigo to form soluble leuco-indigo, which is yellow in color. In other indigo-reducing bacterial cultures, the color of the medium remained unchanged (Fig. 2A), which may have been due to the production of less leuco-indigo by these bacteria.

Colorimetric measurements of leuco-indigo in the culture solution were conducted to quantitatively evaluate the reduction of indigo powder (Fig. 2B). In control cultures without a bacterial inoculation, the concentration of leuco-indigo remained low (<0.3‍ ‍mg L^–1^) during the incubation period. In cultures of known indigo-reducing bacteria, leuco-indigo concentrations were significantly higher than those in the uninoculated control; however, concentrations were still not very high (approximately 2–3‍ ‍mg L^–1^ in cultures of *Am. indicireducens*, *Am. iburiensis*, and *Al. iburiense*, and up to 8.5‍ ‍mg L^–1^ in cultures of *F. fermenti*). In contrast, in the culture of strain TU-1, leuco-indigo concentrations exceeded 10‍ ‍mg L^–1^ on day 2 and reached a maximum of 44.7‍ ‍mg L^–1^ on day 7. Due to the lack of a marked difference in strain TU-1 growth from other indigo reducers under anaerobic conditions (Supplementary Fig. S3B), our results demonstrate that the ability of strain TU-1 to reduce insoluble indigo was superior to that of known indigo-reducing strains.

The leuco-indigo concentration in the strain TU-1 culture decreased on day 10 (Fig. 2B). This phenomenon may be attributed to microbial activities and/or related to the depletion of reducing agents (*e.g.*, amino acids and peptides) in this experiment. This assumption is corroborated by the strain TU-1 growth profile only exhibiting growth during the initial 3 days in the anaerobic PYA medium (Supplementary Fig. S3B). In addition, in practical indigo dyeing processes, nutrients such as wheat bran are typically supplemented monthly or more frequently to prevent a decline in dyeing activity, *i.e.*, to prevent a decline in leuco-indigo concentration (Lopes *et al.*, 2021). These findings suggest that an adequate reducing power supply, and consequently microbial activation, is essential for the long-term maintenance of staining activity. Further experiments involving extended incubation periods and organic matter supplementation during incubations may provide a more detailed understanding of practical indigo dyeing processes.

Although known indigo-reducing strains showed a capacity for indigo reduction when evaluated on an agar medium supplemented with indigo carmine, the reduction ability of strain TU-1 was weak (Supplementary Fig. S2). These results suggest that the reduction capacity for indigo carmine does not correlate with that of insoluble indigo, as proposed by Nakagawa *et al.* (2022). The detailed mole­cular mechanisms underlying microbial indigo reduction remain unclear, and the reasons for reducing property-related differences between insoluble indigo and indigo carmine are unknown. Since insoluble indigo necessitates extracellular reduction, we infer that microorganisms capable of reducing insoluble indigo possess extracellular electron transfer abilities. However, most indigo-reducing bacteria, including those investigated in the present study, are Gram-positive bacteria of the phylum *Bacillota*, and less information is currently available on their extracellular electron transfer than for Gram-negative bacteria. Nicholson and John (2005) reported that the indigo-reducing activity of *Clostridium isatidis* was enhanced by supplementation with electron mediators (*e.g.*, anthraquinone-2,6-disulfonic acid), and the culture supernatants of this bacterium contained unidentified electron mediators. Nakagawa *et al.* (2022) also reported that supplementation with electron mediators (*e.g.*, anthraquinone) promoted the reduction of insoluble indigo by multiple indigo-reducing bacteria. In contrast, Suzuki *et al.* (2018) and Yoneda *et al.* (2020) identified flavin mononucleotide (FMN)-containing enzymes from *Bacillus* spp. that exhibited NADH-dependent indigo carmine-reducing activities, suggesting the existence of an electron mediator-independent direct indigo reduction pathway. However, since these are intracellular enzymes, their contribution to the reduction of insoluble indigo remains unclear. Light *et al.* (2018) reported the extracellular electron transfer mechanism of *Listeria monocytogenes*, a Gram-positive bacterium known for its iron and electrode reduction activities. In *L. monocytogenes*, a cell membrane-anchored cell surface flavoprotein is crucial for its extracellular electron transfer. This flavoprotein accepts intracellular NADH oxidation-derived reducing power and reduces extracellular flavins, which function as electron mediators in iron and electrode reduction. Membrane-bound proteins that transfer electrons to extracellular electron shuttles (such as RnfD, PepSy, and MsrQ) have been described (Méheust *et al.*, 2021). The RnfD gene sequence (complement [92380–93351]) is present in *T. creatinini* strain BN11 (Accession no. SUSS00000000.1; total length 2,611,442 bp), which is a phylogenetic neighboring strain of stain TU-1. Therefore, extracellular electron transfer via mediator compounds is assumed to be the main contributor to the microbial reduction of insoluble indigo. In addition, the ability to excrete mediator compounds and reduce them on cell surfaces are regarded as major factors that enable microorganisms to reduce insoluble indigo.

The 16S rRNA gene of strain TU-1 was highly similar to the predominant sequences retrieved from an alkaline enrichment culture of insoluble iron oxide-reducing bacteria (99.8% identity to uncultured *Tissierella* sp. clone Fe61 [KF362103]) (Fuller *et al.*, 2014). When strain TU-1 was cultured in PYA medium supplemented with poorly crystalline iron oxide, reddish-brown iron oxide particles turned into black precipitates (possibly magnetite and/or iron sulfide) within one week of incubation (Supplementary Fig. S2). Therefore, strain TU-1 may possess the ability to reduce iron oxide, not only insoluble indigo, and exhibits a high capacity for extracellular electron transfer. Fuller *et al.* (2014) detected flavin-like electron mediators in alkaline iron-reducing enrichment cultures dominated by uncultured *Tissierellaceae*. They discussed the involvement of electron mediators in facilitating extracellular electron transfer. Based on these findings, we hypothesized that strain TU-1 produces electron mediator compounds that facilitate extracellular electron transfer to insoluble indigo.

### Effects of electron mediators on insoluble indigo reduction

**Fig. 3.**
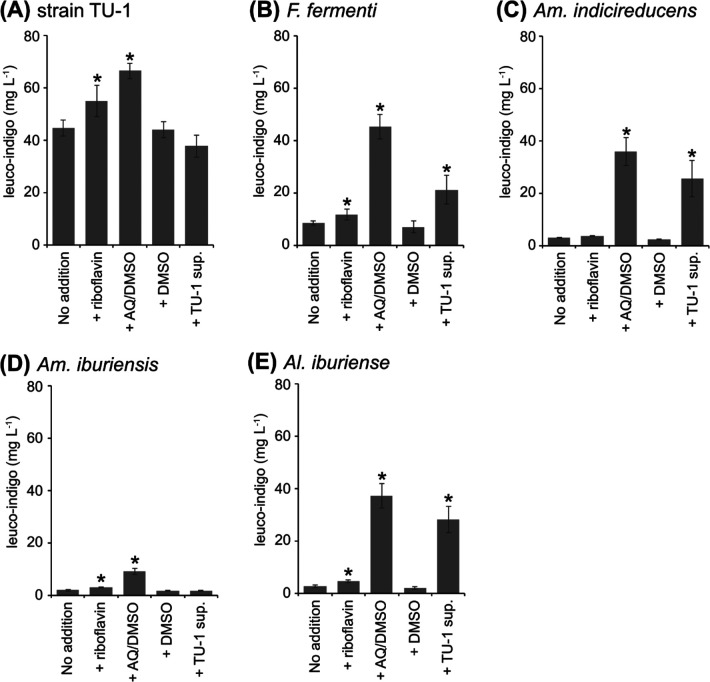
Effects of electron mediator compounds and the culture supernatant of strain TU-1 on the reduction of indigo powder by indigo-reducing strains. (A) Strain TU-1, (B) *Fundicoccus fermenti* JCM 34140^T^, (C) *Amphibacillus indicireducens* JCM 17250^T^, (D) *Amphibacillus iburiensis* JCM 18529^T^, and (E) *Alkalibacterium iburiense* JCM 12662^T^. Each strain was cultured in PYA medium supplemented with 1‍ ‍g‍ ‍L^–1^ of indigo powder. Each culture was subjected to the following culture conditions: +riboflavin: 10‍ ‍mg L^–1^ of riboflavin was supplemented; +AQ/DMSO: 10‍ ‍mg L^–1^ of anthraquinone (AQ) solubilized in dimethyl sulfoxide (DMSO) was supplemented; +DMSO: DMSO was added at the same concentration as the AQ/DMSO condition (2% [v/v]); +TU-1 sup.: 50% of the original volume of PYA medium was reduced by the culture supernatant of strain TU-1. The concentrations of leuco-indigo were quantified on days 0, 2, 4, 7, and 10 for each culture condition and the maximum value is presented in the graphs. Asterisks represent significant increases (*P*<0.05) from the control (No addition). Data are presented as the means of three independent cultures, and error bars represent standard deviations. Culture experiments were conducted in triplicate. The Student’s *t*-test was used to assess the significance of treatment effects.

In the present study, riboflavin and anthraquinone were used as model compounds for water-soluble and -insoluble electron mediators, respectively. Flavin compounds, including riboflavin, serve as cofactors for intracellular enzymes and are frequently secreted extracellularly to perform diverse physiological functions (García-Angulo, 2017). For example, iron oxide-reducing bacteria, such as *Shewanella* spp., have been shown to secrete flavin compounds extracellularly in order to facilitate extracellular electron transfer (Marsili *et al.*, 2008; von Canstein *et al.*, 2008). The supplementation of cultures of strain TU-1 and known indigo-reducing strains with riboflavin (10‍ ‍mg L^–1^) increased leuco-indigo production in all cultures, except for that of *Am. indicireducens*; however, these enhancing effects were not very strong (1.2- to 1.8-fold increases, +riboflavin in Fig. 3).

Hydrophobic electron mediators, such as quinones and phenazines, play a crucial role in microbial extracellular electron transfer (Watanabe *et al.*, 2009). *Shewanella* spp. have been shown to produce quinone-based mediators in order to facilitate extracellular electron transfer (Newman and Kolter, 2000; Mevers *et al.*, 2019). Previous studies reported that the addition of quinone-based mediators enhanced the microbial reduction of insoluble indigo (Nicholson and John, 2005; Nakagawa *et al.*, 2022). In the‍ ‍present study, supplementation with anthraquinone (10‍ ‍mg‍ ‍L^–1^) promoted leuco-indigo production more strongly than the addition of riboflavin to cultures of strain TU-1 and known indigo-reducing bacteria (+AQ/DMSO, Fig. 3). While the reduction-promoting effect was a 1.5-fold increase for strain TU-1, which originally exhibited high reduction activity in the control (no addition, Fig. 3), reduction-promoting effects were as high as 4.7- to 14.4-fold increases for other indigo-reducing bacteria. No reduction-promoting effect was observed in cultures supplemented with DMSO (2% v/v), which was used as a solvent for anthraquinone (+DMSO, Fig. 3). These results suggest that electron mediators promoted the reduction of insoluble indigo and that the reduction-promoting effects of hydrophobic mediators were stronger than those of hydrophilic mediators.

### Effects of the strain TU-1 culture supernatant on insoluble indigo reduction

To confirm the hypothesis of the superior capacity of strain TU-1 to produce electron mediators that facilitate extracellular electron transfer, we assessed the effects of the culture supernatant of strain TU-1 on insoluble indigo reduction (+TU-1 sup., Fig. 3). The addition of the TU-1 culture supernatant did not significantly affect the insoluble indigo reduction activity of strain TU-1 or *Am. iburiensis*. In contrast, the three other species exhibited a significant increase in leuco-indigo production after supernatant supplementation (2.5- to 10.9-fold increase); however, these effects were weaker than those observed following the addition of anthraquinone. These results demonstrate the high capacity of strain TU-1 to extracellularly secrete electron mediators, which represent one of the factors responsible for its high insoluble indigo reduction activity.

Further studies are needed to confirm the types and quantities of mediators produced by strain TU-1 and assess its potential contribution to practical indigo dyeing processes in the future. *Shewanella* spp. may accumulate up to several μM of electron mediators in culture solutions and at even higher concentrations in biofilms (Renslow *et al.*, 2013). Mediator concentrations in biofilms may be comparative to that used in the present study (10‍ ‍mg L^–1^ of anthraquinone is equivalent to 48‍ ‍μM). Since cultures supplemented with half‍ ‍the volume of the TU-1 supernatant exerted similar reduction-promoting effects to those observed in anthraquinone-supplemented cultures, strain TU-1 appears to produce electron mediators at concentrations up to several tens of μM. In addition, since indigo-reducing bacteria are considered to be abundant in the vicinity of precipitated indigo, the mediators produced may accumulate at high concentrations around precipitates.

The mediators produced by strain TU-1 promoted the indigo reduction activity of strain TU-1 as well as that of other indigo reducers isolated from carbohydrate-supplemented media. Although the known indigo-reducing bacteria used in this study exhibited weak insoluble indigo-reducing activities in PYA medium (not with supplemented sugars), these bacteria have often been detected as dominant species in indigo dye suspensions (Okamoto *et al.*, 2017; Tu *et al.*, 2019, 2021; Lopes *et al.*, 2021; Farjana *et al.*, 2023). These bacteria may contribute to indigo reduction by utilizing electron mediators produced by other bacteria. It is also important to note that the effects of the mediator compounds and TU-1 supernatant varied among the four strains of indigo-reducing bacteria exami­ned. *Am. iburiensis* exhibited low insoluble indigo-reducing activity even after the addition of anthraquinone and the TU-1 supernatant, even though its capacity for reducing indigo carmine was similar to that of other indigo reducers. Each indigo-reducing bacterium may exhibit its own preference for mediator compounds, and *Am. iburiensis* may utilize mediators other than those produced by TU-1. In addition to those produced by microorganisms, plant-derived aromatic compounds in *sukumo* are potential candidates for mediators. Chen *et al.* (2021) reported that lignin-derived aromatic compounds acted as electron mediators and facilitated the reductive degradation of azo dyes. Moreover, Nakagawa *et al.* (2025) showed that *sukumo*-derived and commercial lignin both enhanced microbial indigo reduction. Further investigations of the dynamics of mediator species produced by microorganisms, as well as those derived from *sukumo*, in actual dye suspensions will provide a more detailed understanding of the traditional indigo fermentation process. Strain TU-1, which exhibits a different preference for substrates and extracellular electron transfer mechanisms from those of reported indigo-reducing bacteria, may play important roles that differ from those of reported bacteria both in indigo reduction and in the nutrient cycle for this sustainable ecosystem.

## Conclusion

In the present study, we successfully isolated a novel bacterial strain belonging to the family *Tissierellaceae* that exhibited a high reduction capacity for insoluble indigo. Although *Tissierellaceae* bacteria hypothetically contribute to indigo reduction based solely on culture-independent studies, this study provides the first demonstration of this phenomenon using an isolated strain. The isolate obtained, strain TU-1, exhibited weak reducing activity for soluble indigo carmine and stronger activity for insoluble indigo in PYA medium than that of known indigo-reducing bacteria. The present results show that strain TU-1 possessed the‍ ‍ability to extracellularly secrete electron mediators, facilitating indigo reduction not only by strain TU-1 itself, but also by other indigo-reducing bacteria. Further studies on the electron mediators produced by *Tissierellaceae* bacteria, as well as interspecies interactions among microbes in dye suspensions, will enhance our understanding of the mechanisms underlying indigo reduction in practical indigo dyeing processes.

## Citation

Tu, Z., and Yumoto, I. (2025) Isolation of a *Tissierellaceae* Bacterium Exhibiting a High Reduction Potential for Insoluble Indigo Dyes. *Microbes Environ ***40**: ME24104.

https://doi.org/10.1264/jsme2.ME24104

## Supplementary Material

Supplementary Material

## References

[B1] Aino, K., Hirota, K., Matsuno, T., Morita, N., Nodasaka, Y., Fujiwara, T., et al. (2008) *Bacillus polygoni* sp. nov., a moderately halophilic, non-motile obligate alkaliphile isolated from indigo balls. Int J Syst Evol Microbiol 58: 120–124. 18175695 10.1099/ijs.0.65193-0

[B2] Aino, K., Narihiro, T., Minamida, K., Kamagata, Y., Yoshimune, K., and Yumoto, I. (2010) Bacterial community characterization and dynamics of indigo fermentation. FEMS Microbiol Ecol 74: 174–183. 20695891 10.1111/j.1574-6941.2010.00946.x

[B3] Aino, K., Hirota, K., Okamoto, T., Tu, Z., Matsuyama, H., and Yumoto, I. (2018) Microbial communities associated with indigo fermentation that thrive in anaerobic alkaline environments. Front Microbiol 9: 2196. 30279681 10.3389/fmicb.2018.02196 PMC6153312

[B4] Alauzet, C., Marchandin, H., Courtin, P., Mory, F., Lemée, L., Pons, J.L., et al. (2014) Multilocus analysis reveals diversity in the genus *Tissierella*: description of *Tissierella carlieri* sp. nov. in the new class *Tissierellia* classis nov. Syst Appl Microbiol 37: 23–34. 24268443 10.1016/j.syapm.2013.09.007

[B5] Altschul, S.F., Madden, T.L., Schäffer, A.A., Zhang, J., Zhang, Z., Miller, W., and Lipman, D.J. (1997) Gapped BLAST and PSI-BLAST: a new generation of protein database search programs. Nucleic Acids Res 25: 3389–3402. 9254694 10.1093/nar/25.17.3389 PMC146917

[B6] Bellali, S., Haddad, G., Pham, T.P., Iwaza, R., Ibrahim, A., Armstrong, N., et al. (2022) Draft genomes and descriptions of *Urmitella timonensis* gen. nov., sp. nov. and *Marasmitruncus massiliensis* gen. nov., sp. nov., isolated from severely malnourished African children using culturomics. Antonie van Leeuwenhoek 115: 1349–1361. 36149539 10.1007/s10482-022-01777-x PMC9584879

[B7] Chen, R.P., Cai, J.L., Li, Q., Yu, L., Wei, X.Y., Gan, C.H., et al. (2021) Enhancement on the microbial extracellular electron transfers by modified lignin materials: Application on decolorization of azo dye. J Mater Res Technol 15: 5265–5276.

[B8] Farjana, N., Tu, Z., Furukawa, H., and Yumoto, I. (2023) Environmental factors contributing to the convergence of bacterial community structure during indigo reduction. Front Microbiol 14: 1097595. 36876097 10.3389/fmicb.2023.1097595 PMC9978934

[B9] Farrow, J.A., Lawson, P.A., Hippe, H., Gauglitz, U., and Collins, M.D. (1995) Phylogenetic evidence that the gram-negative nonsporulating bacterium *Tissierella* (*Bacteroides*) *praeacuta* is a member of the *Clostridium* subphylum of the gram-positive bacteria and description of *Tissierella creatinini* sp. nov. Int J Syst Bacteriol 45: 436–440. 8590669 10.1099/00207713-45-3-436

[B10] Felsenstein, J. (1985) Confidence limits on phylogenies: an approach using the bootstrap. Evolution 39: 783–791. 28561359 10.1111/j.1558-5646.1985.tb00420.x

[B11] Fuller, S.J., McMillan, D.G., Renz, M.B., Schmidt, M., Burke, I.T., and Stewart, D.I. (2014) Extracellular electron transport-mediated Fe(III) reduction by a community of alkaliphilic bacteria that use flavins as electron shuttles. Appl Environ Microbiol 80: 128–137. 24141133 10.1128/AEM.02282-13 PMC3910996

[B12] García-Angulo, V.A. (2017) Overlapping riboflavin supply pathways in bacteria. Crit Rev Microbiol 43: 196–209. 27822970 10.1080/1040841X.2016.1192578

[B13] Harms, C., Schleicher, A., Collins, M.D., and Andreesen, J.R. (1998) *Tissierella creatinophila* sp. nov., a gram-positive, anaerobic, non-spore-forming, creatinine-fermenting organism. Int J Syst Bacteriol 48: 983–993. 9734055 10.1099/00207713-48-3-983

[B14] Hirota, K., Aino, K., Nodasaka, Y., Morita, N., and Yumoto, I. (2013a) *Amphibacillus indicireducens* sp. nov., an alkaliphile that reduces an indigo dye. Int J Syst Evol Microbiol 63: 464–469. 22493173 10.1099/ijs.0.037622-0

[B15] Hirota, K., Aino, K., Nodasaka, Y., and Yumoto, I. (2013b) *Oceanobacillus indicireducens* sp. nov., a facultative alkaliphile that reduces an indigo dye. Int J Syst Evol Microbiol 63: 1437–1442. 22843722 10.1099/ijs.0.034579-0

[B16] Hirota, K., Aino, K., and Yumoto, I. (2013c) *Amphibacillus iburiensis* sp. nov., an alkaliphile that reduces an indigo dye. Int J Syst Evol Microbiol 63: 4303–4308. 23832971 10.1099/ijs.0.048009-0

[B17] Hirota, K., Hanaoka, Y., Nodasaka, Y., and Yumoto, I. (2013d) *Oceanobacillus polygoni* sp. nov., a facultatively alkaliphile isolated from indigo fermentation fluid. Int J Syst Evol Microbiol 63: 3307–3312. 23504965 10.1099/ijs.0.048595-0

[B18] Hirota, K., Aino, K., and Yumoto, I. (2016a) *Fermentibacillus polygoni* gen. nov., sp. nov., an alkaliphile that reduces indigo dye. Int J Syst Evol Microbiol 66: 2247–2253. 26971318 10.1099/ijsem.0.001015

[B19] Hirota, K., Okamoto, T., Matsuyama, H., and Yumoto, I. (2016b) *Polygonibacillus indicireducens* gen. nov., sp. nov., an indigo-reducing and obligate alkaliphile isolated from indigo fermentation liquor for dyeing. Int J Syst Evol Microbiol 66: 4650–4656. 27503611 10.1099/ijsem.0.001405

[B20] Hirota, K., Nishita, M., Matsuyama, H., and Yumoto, I. (2017) *Paralkalibacillus indicireducens* gen., nov., sp. nov., an indigo-reducing obligate alkaliphile isolated from indigo fermentation liquor used for dyeing. Int J Syst Evol Microbiol 67: 4050–4056. 28905696 10.1099/ijsem.0.002248

[B21] Hirota, K., Nishita, M., Tu, Z., Matsuyama, H., and Yumoto, I. (2018) *Bacillus fermenti* sp. nov., an indigo-reducing obligate alkaliphile isolated from indigo fermentation liquor for dyeing. Int J Syst Evol Microbiol 68: 1123–1129. 29458563 10.1099/ijsem.0.002636

[B22] Huang, X., Dong, W., Wang, H., and Feng, Y. (2018) Role of acid/alkali-treatment in primary sludge anaerobic fermentation: Insights into microbial community structure, functional shifts and metabolic output by high-throughput sequencing. Bioresour Technol 249: 943–952. 29145121 10.1016/j.biortech.2017.10.104

[B23] Kato, S., Haruta, S., Cui, Z.J., Ishii, M., Yokota, A., and Igarashi, Y. (2004) *Clostridium straminisolvens* sp. nov., a moderately thermophilic, aerotolerant and cellulolytic bacterium isolated from a cellulose-degrading bacterial community. Int J Syst Evol Microbiol 54: 2043–2047. 15545431 10.1099/ijs.0.63148-0

[B24] Li, D.H., Abuduaini, R., Du, M.X., Wang, Y.J., Chen, H.H., Zhou, N., et al. (2022) *Alkaliphilus flagellatus* sp. nov., *Butyricicoccus intestinisimiae* sp. nov., *Clostridium mobile* sp. nov., *Clostridium simiarum* sp. nov., *Dysosmobacter acutus* sp. nov., *Paenibacillus brevis* sp. nov., *Peptoniphilus ovalis* sp. nov. and *Tissierella simiarum* sp. nov., isolated from monkey faeces. Int J Syst Evol Microbiol 72: 005276. 35258450 10.1099/ijsem.0.005276 PMC9558573

[B25] Light, S.H., Su, L., Rivera-Lugo, R., Cornejo, J.A., Louie, A., Iavarone, A.T., et al. (2018) A flavin-based extracellular electron transfer mechanism in diverse Gram-positive bacteria. Nature 562: 140–144. 30209391 10.1038/s41586-018-0498-z PMC6221200

[B26] Logan, M., Zhu, F., Lens, P.N.L., and Cetecioglu, Z. (2023) Influence of pH, heat treatment of inoculum, and selenium oxyanions on concomitant selenium bioremediation and volatile fatty acid production from food waste. ACS Omega 8: 34397–34409. 37779932 10.1021/acsomega.2c06459 PMC10535259

[B27] Lopes, H.F.S., Tu, Z., Sumi, H., and Yumoto, I. (2021) Analysis of bacterial flora of indigo fermentation fluids utilizing composted indigo leaves (*sukumo*) and indigo extracted from plants (Ryukyu-ai and Indian indigo). J Biosci Bioeng 132: 279–286. 34127379 10.1016/j.jbiosc.2021.05.004

[B28] Lovley, D.R., and Phillips, E.J. (1986) Organic matter mineralization with reduction of ferric iron in anaerobic sediments. Appl Environ Microbiol 51: 683–689. 16347032 10.1128/aem.51.4.683-689.1986 PMC238947

[B29] Marsili, E., Baron, D.B., Shikhare, I.D., Coursolle, D., Gralnick, J.A., and Bond, D.R. (2008) *Shewanella* secretes flavins that mediate extracellular electron transfer. Proc Natl Acad Sci U S A 105: 3968–3973. 18316736 10.1073/pnas.0710525105 PMC2268775

[B30] Maspolim, Y., Zhou, Y., Guo, C., Xiao, K., and Ng, W.J. (2015) The effect of pH on solubilization of organic matter and microbial community structures in sludge fermentation. Bioresour Technol 190: 289–298. 25965254 10.1016/j.biortech.2015.04.087

[B31] Méheust, R., Huang, S., Rivera-Lugo, R., Banfield, J.F., and Light, S.H. (2021) Post-translational flavinylation is associated with diverse extracytosolic redox functionalities throughout bacterial life. eLife 10: e66878. 34032212 10.7554/eLife.66878 PMC8238504

[B32] Mevers, E., Su, L., Pishchany, G., Baruch, M., Cornejo, J., Hobert, E., et al. (2019) An elusive electron shuttle from a facultative anaerobe. eLife 8: e48054. 31232690 10.7554/eLife.48054 PMC6687433

[B33] Milanović, V., Osimani, A., Taccari, M., Garofalo, C., Butta, A., Clementi, F., and Aquilanti, L. (2017) Insight into the bacterial diversity of fermentation woad dye vats as revealed by PCR-DGGE and pyrosequencing. J Ind Microbiol Biotechnol 44: 997–1004. 28246965 10.1007/s10295-017-1921-4

[B34] Nakagawa, K., Takeuchi, M., Tada, M., Matsunaga, M., Kugo, M., Kiyofuji, S., et al. (2022) Isolation and characterization of indigo-reducing bacteria and ana­lysis of microbiota from indigo fermentation suspensions. Biosci Biotechnol Biochem 86: 273–281. 34864880 10.1093/bbb/zbab209

[B35] Nakagawa, K., Ohata, H., Takeuchi, M., Matsunaga, M., Sowa, K., Sakamoto, T., et al. (2025) Effects of lignin on indigo-reducing activity and indigo particle size in indigo dye suspensions. Biosci Biotechnol Biochem 89: 141–144. 10.1093/bbb/zbae151 39589215

[B36] Nakajima, K., Hirota, K., Nodasaka, Y., and Yumoto, I. (2005) *Alkalibacterium iburiense* sp. nov., an obligate alkaliphile that reduces an indigo dye. Int J Syst Evol Microbiol 55: 1525–1530. 16014476 10.1099/ijs.0.63487-0

[B37] Nazina, T.N., Bidzhieva, S.K., Grouzdev, D.S., Sokolova, D.S., Tourova, T.P., Parshina, S.N., et al. (2020) *Soehngenia longivitae* sp. nov., a fermenting bacterium isolated from a petroleum reservoir in Azerbaijan, and emended description of the genus *Soehngenia*. Microorganisms 8: 1967. 33322329 10.3390/microorganisms8121967 PMC7763609

[B38] Newman, D.K., and Kolter, R. (2000) A role for excreted quinones in extracellular electron transfer. Nature 405: 94–97. 10811225 10.1038/35011098

[B39] Nicholson, S.K., and John, P. (2005) The mechanism of bacterial indigo reduction. Appl Microbiol Biotechnol 68: 117–123. 15635460 10.1007/s00253-004-1839-4

[B40] Okamoto, T., Aino, K., Narihiro, T., Matsuyama, H., and Yumoto, I. (2017) Analysis of microbiota involved in the aged natural fermentation of indigo. World J Microbiol Biotechnol 33: 70. 28285451 10.1007/s11274-017-2238-1

[B41] Park, S., Ryu, J.Y., Seo, J., and Hur, H.G. (2012) Isolation and characterization of alkaliphilic and thermotolerant bacteria that reduce insoluble indigo to soluble leuco-indigo from indigo dye vat. J Korean Soc Appl Biol Chem 55: 83–88.

[B42] Renslow, R., Babauta, J., Kuprat, A., Schenk, J., Ivory, C., Fredrickson, J., and Beyenal, H. (2013) Modeling biofilms with dual extracellular electron transfer mechanisms. Phys Chem Chem Phys 15: 19262–19283. 24113651 10.1039/c3cp53759e PMC3868370

[B43] Saitou, N., and Nei, M. (1987) The neighbor-joining method: a new method for reconstructing phylogenetic trees. Mol Biol Evol 4: 406–425. 3447015 10.1093/oxfordjournals.molbev.a040454

[B44] Suzuki, H., Abe, T., Doi, K., and Ohshima, T. (2018) Azoreductase from alkaliphilic *Bacillus* sp. AO1 catalyzes indigo reduction. Appl Microbiol Biotechnol 102: 9171–9181. 30105570 10.1007/s00253-018-9284-y

[B45] Tamura, K., Stecher, G., and Kumar, S. (2021) MEGA11: Molecular Evolutionary Genetics Analysis Version 11. Mol Biol Evol 38: 3022–3027. 33892491 10.1093/molbev/msab120 PMC8233496

[B46] Tu, Z., Lopes, H.F.S., Igarashi, K., and Yumoto, I. (2019) Characterization of the microbiota in long- and short-term natural indigo fermentation. J Ind Microbiol Biotechnol 46: 1657–1667. 31432338 10.1007/s10295-019-02223-0

[B47] Tu, Z., Lopes, H.F.S., Narihiro, T., and Yumoto, I. (2021) The mechanism underlying of long-term stable indigo reduction state in indigo fermentation using *sukumo* (composted *Polygonum tinctorium* leaves). Front Microbiol 12: 698674. 34367099 10.3389/fmicb.2021.698674 PMC8342947

[B48] Tu, Z., Lopes, H.F.S., and Yumoto, I. (2022) *Fundicoccus fermenti* sp. nov., an indigo-reducing facultative anaerobic alkaliphile isolated from indigo fermentation liquor used for dyeing. Int J Syst Evol Microbiol 72: 005239. 10.1099/ijsem.0.005239 35156919

[B49] Ueno, Y. (2023) Indigo dyeing and fermentation sukumo, essential for traditional Japanese aizome. Art Sci 7: 1–7.

[B50] von Canstein, H., Ogawa, J., Shimizu, S., and Lloyd, J.R. (2008) Secretion of flavins by *Shewanella* species and their role in extracellular electron transfer. Appl Environ Microbiol 74: 615–623. 18065612 10.1128/AEM.01387-07 PMC2227709

[B51] Watanabe, K., Manefield, M., Lee, M., and Kouzuma, A. (2009) Electron shuttles in biotechnology. Curr Opin Biotechnol 20: 633–641. 19833503 10.1016/j.copbio.2009.09.006

[B52] Wu, K., Dai, L., Tu, B., Zhang, X., Zhang, H., Deng, Y., et al. (2020) *Gudongella oleilytica* gen. nov., sp. nov., an aerotorelant bacterium isolated from Shengli oilfield and validation of family *Tissierellaceae*. Int J Syst Evol Microbiol 70: 951–957. 31751197 10.1099/ijsem.0.003854

[B53] Wylensek, D., Hitch, T.C.A., Riedel, T., Afrizal, A., Kumar, N., Wortmann, E., et al. (2020) A collection of bacterial isolates from the pig intestine reveals functional and taxonomic diversity. Nat Commun 11: 6389. 33319778 10.1038/s41467-020-19929-w PMC7738495

[B54] Yoneda, K., Yoshioka, M., Sakuraba, H., Araki, T., and Ohshima, T. (2020) Structural and biochemical characterization of an extremely thermostable FMN-dependent NADH-indigo reductase from *Bacillus smithii*. Int J Biol Macromol 164: 3259–3267. 32861785 10.1016/j.ijbiomac.2020.08.197

[B55] Yumoto, I., Hirota, K., Nodasaka, Y., Yokota, Y., Hoshino, T., and Nakajima, K. (2004) *Alkalibacterium psychrotolerans* sp. nov., a psychrotolerant obligate alkaliphile that reduces an indigo dye. Int J Syst Evol Microbiol 54: 2379–2383. 15545487 10.1099/ijs.0.63130-0

[B56] Yumoto, I., Hirota, K., Nodasaka, Y., Tokiwa, Y., and Nakajima, K. (2008) *Alkalibacterium indicireducens* sp. nov., an obligate alkaliphile that reduces indigo dye. Int J Syst Evol Microbiol 58: 901–905. 18398191 10.1099/ijs.0.64995-0

