# Supplementary material for: Isolation of a Tissierellaceae Bacterium Exhibiting a High Reduction Potential for Insoluble Indigo Dyes
Source: Microbes Environ. 2025 Jul 12;40(3):ME24104. doi: 10.1264/jsme2.ME24104 (PMC12501876; doi:10.1264/jsme2.ME24104)

Supplementary Information

Isolation of a *Tissierellaceae* bacterium exhibiting a high reduction potential for insoluble indigo dyes

Zhihao Tu[1], Isao Yumoto[2]*

[1]*MOE Key Laboratory of Groundwater Circulation and Evolution & School of Water Resources and Environment, China University of Geosciences (Beijing), No.29, Xueyuan Road, Haidian District, Beijing 100083, PR China*; [2]*Institute for Open and Transdisciplinary Research Initiatives, Osaka University, 2-1 Yamada-oka, Osaka, 565-0871, Japan*.

*Corresponding author: E-mail: yumoto.isao.atr@osaka-u.ac.jp; Tel.: (+81) 6 6879 7382; Fax: (+81) 6 6879 7454

Figs. S1-S2

Fig. S1. The standard curve used for quantification of indigo. The absorbance at 620 nm of indigo solutions serially diluted with dimethyl sulfoxide (DMSO) were measured. An approximation curve ($y = 0.082x$, $r^2 = 0.99$) derived from the least-squares method is presented as a dashed line.

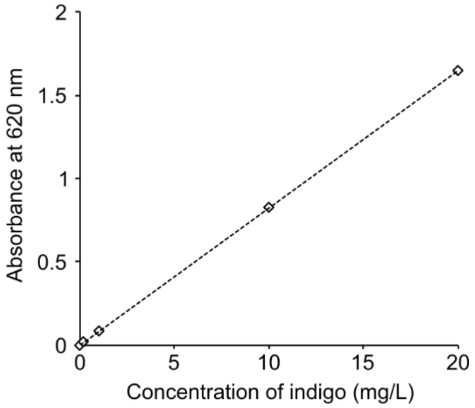

Fig. S2. Reduction of indigo carmine by strain TU-1 and known indigo-reducing bacteria. Each strain was inoculated on PYA (peptone/yeast extract/alkaline) agar medium supplemented with 2 g l^{-1} of indigo carmine. Photographs were taken after incubation under anaerobic conditions. The period of incubation is indicated in the figure panels. *F. fermenti*, *Fundicoccus fermenti* JCM 34140^{T}; *Am. indicireducens*, *Amphibacillus indicireducens* JCM 17250^{T}; *Am. iburiensis*, *Amphibacillus iburiensis* JCM 18529^{T}; *Al. iburiense*, *Alkalibacterium iburiense* JCM 12662^{T}.

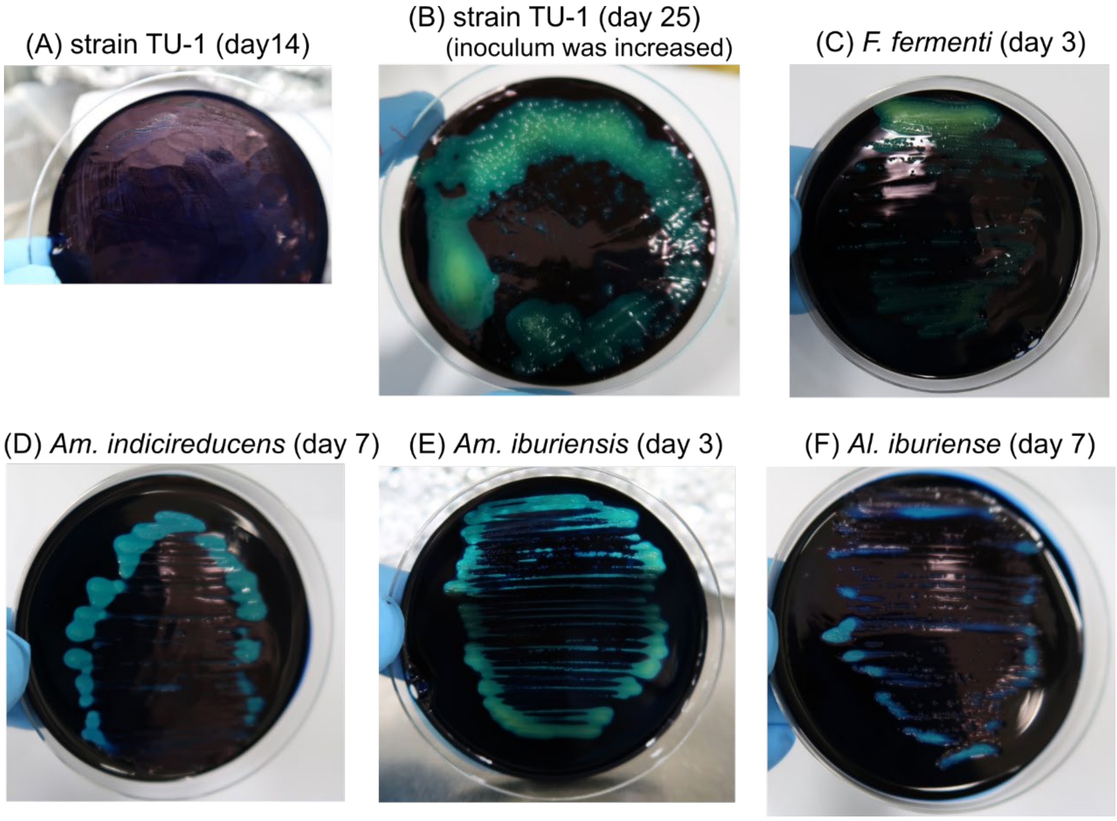

Fig. S3. Growth properties of the microorganisms used in this study. Strain TU-1, *Fundicoccus fermenti*, *Amphibacillus indicireducens*, *Amphibacillus iburiensis*, and *Alkalibacterium iburiense* were cultured in peptone/yeast extract/alkaline (PYA) medium under aerobic (A) or anaerobic (B) conditions. These strains were cultured under aerobic and anaerobic conditions at 30°C with (180 rpm) and without shaking, respectively. The gas phase of the anaerobic condition was replaced by nitrogen. Their optical densities at 600 nm (OD_{600}) were measured using uninoculated PYA medium serving as the control. Data are presented as the means of three independent cultures, and error bars represent standard deviations.

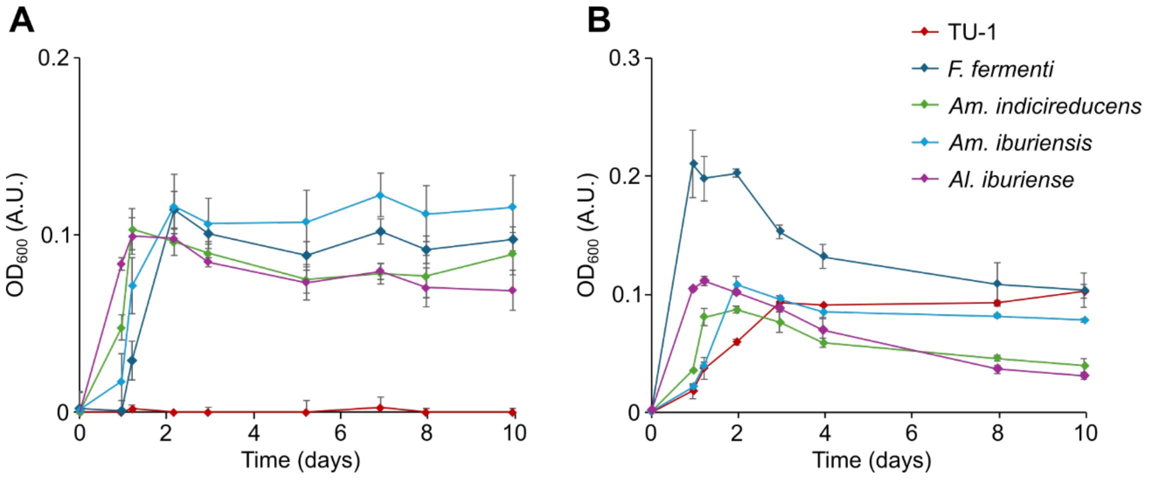

Fig. S4. Reduction of poorly crystalline iron oxides by strain TU-1. Strain TU-1 was cultured in the PYA medium supplemented with 1 mM of poorly crystalline iron oxides. The photograph was taken after 14 days incubation.

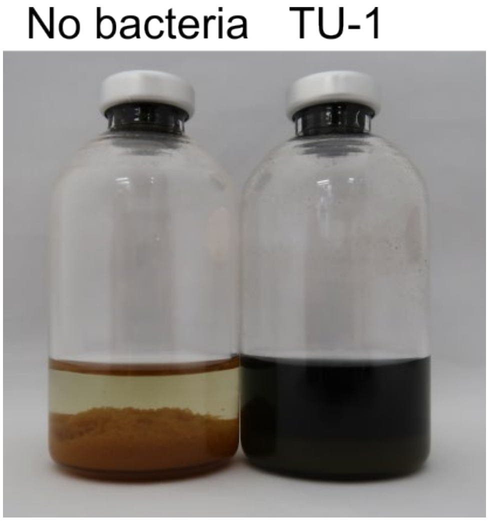

Supplement: Supplementary file 1 — Supplementary Material [file 40_24104_s1.pdf]
